# Clinical investigations for SUS, the Brazilian public health system

**DOI:** 10.1590/S1516-31802012000300008

**Published:** 2012-07-12

**Authors:** Ana Patrícia de Paula, Silvana Pereira Giozza, Michelle Zanon Pereira, Patrícia Souza Boaventura, Leonor Maria Pacheco Santos, Camile Giaretta Sachetti, César Omar Carranza Tamayo, Clarissa Campos Guaragna Kowalski, Flavia Tavares Silva Elias, Suzanne Jacob Serruya, Reinaldo Guimarães

**Affiliations:** I MD, PhD. Coordinator, Postgraduate Program, Escola Superior de Ciências da Saúde, Fundação de Ensino e Pesquisa em Ciências da Saúde, Federal District Health Department, Brasília, Federal District, Brazil.; II DDS, PhD. Consultant, Department of Science and Technology, Ministry of Health, Brasília, Federal District, Brazil.; III MSc. Consultant Nutritionist, Department of Science and Technology, Ministry of Health, Brasília, Federal District, Brazil.; IV Consultant Specialist Pharmacist, Department of Science and Technology, Ministry of Health, Brasília, Federal District, Brazil.; V PhD. Chemist and Professor, Department of Public Health, Universidade de Brasília (UnB), Brasília, Federal District, Brazil.; VI MSc. Consultant Pharmacist, Department of Science and Technology, Ministry of Health, Brasília, Federal District, Brazil.; VII MD, PhD. Consultant, Department of Science and Technology, Ministry of Health, Brasília, Federal District, Brazil.; VIII DDS, MSc. Consultant, Department of Science and Technology, Ministry of Health, Brasília, Federal District, Brazil.; IX MSc. Nutritionist, General Coordinator, Department of Science and Technology, Ministry of Health, Brasília, Federal District, Brazil.; X MD, PhD. Consultant, Latin American Centre for Perinatology and Women and Reproductive Health, Pan-American Health Organization, Montevideo, Uruguay.; XI MD, MSc. Professor, Universidade Estadual do Rio de Janeiro (UERJ), Rio de Janeiro, Brazil.

**Keywords:** Biomedical research, Technology assessment, biomedical, Health policy, Health services research, Public health, Pesquisa biomédica, Avaliação da tecnologia biomédica, Política de saúde, Pesquisa sobre serviços de saúde, Saúde pública

## Abstract

**CONTEXT AND OBJECTIVE:**

Scientific and technological development is crucial for advancing the Brazilian health system and for promoting quality of life. The way in which the Brazilian Ministry of Health has supported clinical research to provide autonomy, self-sufficiency, competitiveness and innovation for the healthcare industrial production complex, in accordance with the National Policy on Science, Technology and Innovation in Healthcare, was analyzed.

**DESIGN AND SETTING:**

Descriptive investigation, based on secondary data, conducted at the Department of Science and Technology, Ministry of Health.

**METHODS:**

The Ministry of Health’s research management database, PesquisaSaúde, was analyzed from 2002 to 2009, using the key word “clinical research” in the fields “primary sub-agenda” or “secondary sub-agenda”. The 368 projects retrieved were sorted into six categories: basic biomedical research, preclinical studies, expanded clinical research, clinical trials, infrastructure support and health technology assessment. From a structured review on “clinical research funding”, results from selected countries are presented and discussed.

**RESULTS:**

The amount invested was R$ 140 million. The largest number of projects supported “basic biomedical research”, while the highest amounts invested were in “clinical trials” and “infrastructure support”. The southeastern region had the greatest proportion of projects and financial resources. In some respects, Brazil is ahead of other BRICS countries (Russia, India, China and South Africa), especially with regard to establishing a National Clinical Research Network.

**CONCLUSION:**

The Ministry of Health ensured investments to encourage clinical research in Brazil and contributed towards promoting cohesion between investigators, health policies and the healthcare industrial production complex.

## RESUMO

**CONTEXTO E OBJETIVO:**

O desenvolvimento científico e tecnológico é crucial para avançar o Sistema Único de Saúde e promover qualidade de vida. Analisou-se como o Ministério da Saúde (MS) apoiou a pesquisa clínica para proporcionar autonomia, auto-suficiência, competitividade e inovação no complexo industrial produtivo da saúde, segundo a Política Nacional de Ciência, Tecnologia e Inovação em Saúde.

**TIPO DE ESTUDO E LOCAL:**

Estudo descritivo baseado em dados secundários, realizado no Departamento de Ciência e Tecnologia, Ministério da Saúde.

**MÉTODOS:**

O banco de dados gerencial de pesquisas do MS, PesquisaSaúde, foi analisado de 2002 a 2009, empregando a palavra chave “pesquisa clínica” nos campos “sub-agenda principal” ou “sub-agenda secundária”. Foram encontrados 368 projetos, classificados em seis categorias: pesquisa biomédica básica, estudos pré-clínicos, pesquisa clínica expandida, ensaios clínicos, infraestrutura e avaliação de tecnologias em saúde. A partir da revisão estruturada sobre “financiamento da pesquisa clínica”, resultados de países selecionados são apresentados e discutidos.

**RESULTADOS:**

O total investido foi R$ 140 milhões. A maioria dos projetos apoiou “pesquisa biomédica básica” e os maiores investimentos foram em “ensaios clínicos” e projetos de “infra-estrutura”. O Sudeste deteve a maior proporção de projetos e recursos financeiros. Em alguns aspectos, o Brasil está à frente de outros países do BRICS (Rússia, Índia, China e África do Sul), sobretudo no estabelecimento da Rede Nacional de Pesquisa Clínica.

**CONCLUSÃO:**

O MS assegurou investimentos para incentivar a pesquisa clínica no Brasil, contribuindo para promover a coesão entre os investigadores, as políticas de saúde e o complexo industrial da saúde.

## INTRODUCTION

Scientific and technological development, guided by standards of excellence, ethics and equity, is crucial for advancing the Brazilian Unified Health System (Sistema Único de Saúde, SUS) and for improving quality of life. Article 200, paragraph V of the Brazilian Constitution describes one of the missions of SUS as “to increase scientific and technological development in its area of operation”.^1^ Integration between government activities, universities and businesses in the modern systems of science, technology and innovation that exist in industrialized countries has virtually eliminated the gap between discovery and application. This has also created a “virtuous circle” of positive feedback between research and development and socioeconomic status.^2^

## OBJECTIVE

This article aimed to analyze how the Brazilian Ministry of Health (Ministério da Saúde, MS) has supported national clinical research to provide technological training, autonomy and self-sufficiency, competitiveness and innovation for the healthcare industrial production complex, in accordance with the National Policy on Science, Technology and Innovation in Healthcare.

### The National Agenda of Priorities in Healthcare Research

Between 2002 and 2009, the Department of Science and Technology (Departamento de Ciência e Tecnologia, Decit) of the Secretariat of Science, Technology and Strategic Inputs, at the Ministry of Health, invested almost R$ 693 million to support healthcare research and development. These figures are incremental to those coming from other institutions, notably the Ministry of Science and Technology (Ministério de Ciência e Tecnologia, MCT) and the state research support foundations (Fundação de Apoio à Pesquisa, FAP). The actions were guided by a conceptual framework in which there was an effort to translate these findings into explicit policy formulations for SUS. The National Policy for Science, Technology and Innovation in Healthcare and the National Agenda of Priorities in Healthcare Research (Agenda Nacional de Prioridades de Pesquisa em Saúde, ANPPS) were agreed upon and established through a democratic process involving participation by government officials, researchers, healthcare managers and SUS clients during the Second National Conference on Science, Technology and Innovation in Healthcare.^3^

The National Agenda was organized into 24 sub-agendas, and one of these was dedicated to “clinical research”. This sub-agenda was organized into two sections. The first was broad, and dealt with “General development of clinical research”, including the following items: evaluation of diagnostic methods for therapeutic interventions with new technologies and their applicability; clinical trials on diagnostic and therapeutic procedures originating from national research; and studies to draw up and validate clinical protocols, including those for homeopathic and acupuncture treatments.^4^ The other section described some “Specific themes”, as follows: (i) clinical diagnostic study of congenital diseases, including kinship analysis; (ii) identification of genes, genetic polymorphism and compilation of a genetic database; (iii) clinical evaluation of new generic drugs; (iv) clinical trials on substitutes for high-priced imported drugs or medical devices; (v) clinical trials on complementary therapeutic practices, such as using acupuncture in addition to the usual care to help lessen pain; (vi) clinical evaluation of long-term medication for the most prevalent conditions; (vii) preclinical and clinical research on medicinal plants, phytotherapeutic and bioactive products traditionally used by the population; (viii) cell therapy, stem cells and pharmacogenetics; (ix) application of molecular biology techniques to diagnoses (like serum tests); and (x) clinical evaluation of interventions within physiotherapy, occupational therapy and speech therapy.^4^

### The National Clinical Research Network

Over the last decade, a large number of clinical research protocols have been in progress in Brazil, but the vast majority reflected the priorities of the private laboratories that commissioned them, without devoting space to SUS priorities. The participation of Brazilian researchers was usually restricted to gathering data and running protocols developed in other countries, while analysis and ownership of the data were left entirely to the commissioning companies. Establishment of a clinical research network was devised to place the country in a situation of greater autonomy to develop its own strategic clinical investigations. Considering the priority attributed to the national industrial policy and the need to encourage national production of drugs, vaccines, diagnostic kits and medical devices, it was necessary to develop clinical research platforms.

Within this context, the National Clinical Research Network (Rede Nacional de Pesquisa Clínica, RNPC) was created in 2005, through a Decit initiative, co-financed by the Financier of Studies and Projects (Financiadora de Estudos e Projetos/Ministério da Ciência e Tecnologia, FINEP/MCT), in order to establish a model for clinical research directed towards meeting the needs of SUS. The importance of encouraging and supporting this clinical research network in teaching hospitals lay in the possibility of bringing together national reference centers and experts on certain research topics and procedures. This enabled the cooperation necessary to leverage and accelerate a return of benefits to society through increased knowledge, techniques and products that met the population’s healthcare requirements and strengthened the health technology industrial sector.

Currently, the RNPC is composed of 32 clinical research centers linked to universities spread across the country’s five geographical regions.^5^ The Ministry of Health’s strategy was to create a network aimed primarily at promoting the implementation of all phases of clinical trials on drugs, procedures, equipment, medical devices and new diagnostic methods that were of interest to SUS.^5^ The RNPC undertakes various actions to promote research and contributes to the development of human resources, through offering training courses on clinical research. During the five years since its inception, the network has promoted a great amount of cooperation and exchange. The greater proximity of research centers has allowed the RNPC to promote transfers of technology and knowledge between the research centers and to develop multicenter studies of relevance to the technical and scientific development of SUS, while always guided by the ANPPS.

### Definitions of Clinical Research

There are several characterizations of clinical research within the healthcare literature. The publication resulting from the Second National Conference on Science, Technology and Innovation in Healthcare defined clinical research as the type of research that follows the scientific methodology applicable to humans, known as volunteers or “research subjects”, who may be healthy or sick, according to the aims of such studies.^6^

The Association of American Medical Colleges Task Force on Clinical Research has also adopted a broad definition of the term: “a component of medical and health research intended to produce knowledge essential for understanding human disease, preventing and treating illness and promoting health”.^7^

The European Medicines Agency (EMEA) has defined clinical research as “research on humans, in order to discover or verify the pharmacodynamic, pharmacological, clinical and/or other effects of the product and/or identify adverse reactions to the product under investigation, with the purpose of ascertaining its safety and/or efficacy”.^8^

Some authors have introduced the term expanded clinical research. This type of research activity aims to develop new knowledge and technologies for prevention and diagnosis of disease, and for treatment and rehabilitation of patients; innovations in the provision of healthcare and surveillance services; anthropological studies; new approaches in providing care, surveillance and rehabilitation; and interdisciplinary approaches towards emerging diseases, among others.^9^ The diverse definitions for the term clinical research have to be borne in mind when analyzing clinical investigations developed in Brazil.

## METHODS

This empirical study was based on secondary data obtained from the Decit management database system (PesquisaSaúde).^10^ The system provides information about projects funded by the Ministry of Health and its partners: the MCT, FAPs and National Bank for Economic and Social Development (Banco Nacional de Desenvolvimento Econômico e Social, BNDES). The electronic search covered the years 2002 to 2009, using the key word “clinical research” in the fields “primary sub-agenda” or “secondary sub-agenda”. Projects in the database were classified as “clinical research” either by the principal investigator (PI) of the project or by Decit staff. The process retrieved 368 projects, for which the following information was available: title, abstract, key words, type of research, nature and sector of application, PI name and institution, year, resources approved, types of support, etc.

The projects were then sorted into the following categories: (a) Basic biomedical research, which included experimental or theoretical studies that contribute towards understanding phenomena and observable facts or theories, without immediate use or application;^11^ (b) Preclinical studies, corresponding to application of new molecules in animals, after in vitro trials have shown therapeutic potential, in order to obtain preliminary information on the pharmacological activity and safety of these new molecules; this type of study would also be used to evaluate equipment or healthcare devices on animals;^12^ (c) Expanded clinical research, as defined by Marzochi^9^, except for clinical trials, which were analyzed in a separate category; (d) Clinical trials, including randomized clinical trials (RCTs), which are conducted mainly in developing new drugs,^13^ and are classified into four stages (I to IV);^14^ (e) Infrastructure support, which was fundamental for setting up the National Clinical Research Network; and (f) Health technology assessment (HTA) projects. Some projects were not classified into these categories, due to insufficient information.

The results obtained through some research projects funded by DECIT are concisely presented throughout the text, in order to illustrate the range of projects funded by the Ministry of Health, within the contexts of SUS and the ANPPS.

A structured review was conducted by searching Medline and Embase from 2005 until July 21, 2011. The query “(“clinical” AND “research” AND “funding”)” yielded 680 articles. The titles and abstracts were manually inspected for relevance; 56 papers were acquired and carefully examined; 11 published papers were used in this review. Some results relating to clinical research funding in two developed countries (United States and United Kingdom) and four developing countries (Russia, India, China and South Africa, which together with Brazil form the BRICS group) are presented and discussed.

## RESULTS

An amount of almost R$ 140 million was assigned to 368 projects over the course of the time period studied. Table 1 summarizes the investments made by Decit and partners within the field of clinical research. National Calls for Proposals was the mechanism most used, followed by state calls. The meritocratic open bidding process predominated, accounting for 97% of all the projects financed. Only 12 strategic projects were contracted without bids. The average investment was higher in the national Calls for Proposals, because this category included most of the clinical trials and all of the infrastructure projects.

**Table 1.** Investments by the Ministry of Health and its partners in clinical investigations and infrastructure for clinical research. Brazil, 2002-2009

Description	Type of financial support and selection process			
	State-level calls for proposals	National calls for proposals	Direct contracts	Total
Number of projects	144	212	12	368
Investments (R$)	R$ 11,743,849.65	R$ 123,039,840.20	R$ 4,586,960.47	R$ 139,370,650.30
Investment per project (R$)	R$ 81,554.51	R$ 580,376.60	R$ 382,246.71	R$ 378,724.59

We retrieved 368 projects from the database, but 15 were impossible to categorize; the remaining 353 were classified into six categories (Table 2). Although the largest number of projects was found in the group Basic biomedical research (n = 118), the highest amount of investment was directed towards financing clinical trials (R$ 44 million) and infrastructure projects (R$ 37 million). However, from analyzing the amount allocated per project, infrastructure came first, with about R$ 1.5 million invested per clinical research center, and clinical trials came next, with an average of R$ 0.84 million per investigation.

**Table 2.** Investments by the Ministry of Health and its partners in clinical investigations and infrastructure for clinical research, according to project category. Brazil, 2002-2009

Project category	Number of projects	Proportion of projects	Funds invested per category (R$)	Proportion of funds	Funds invested per project (R$)
Basic biomedical	118	32.1%	22,026,573.30	15.8%	186,665.88
Preclinical	58	15.8%	10,337,452.40	7.4%	178,231.94
Expanded clinical	99	26.9%	18,931,425.74	13.6%	191,226.52
Clinical trials	53	14.4%	44,480,371.51	31.9%	839,252.29
Infrastructure	24	6.5%	37,339,604.73	26.8%	1,555,816.86
HTA projects*	1	0.3%	386,851.52	0.3%	386,851.52
Not classified	15	4.1%	5,868,371.12	4.2%	391,224.74
Total	368	100%	139,370,650.32	100%	378,724.59

HTA = Health Technology Assessment; *HTA projects are classified in a specific HTA sub-agenda; over the study period, 294 HTA projects were financed, which, however, are beyond the scope of the present study.

We concluded that the projects supported over the reporting period covered almost 94% of the topics included in the ANPPS clinical research sub-agenda previously described.^4^ The fields without financial support were those relating to: (v) clinical trials on complementary therapeutic practices, such as acupuncture; (vi) clinical evaluation of long-term medication for the most prevalent conditions; and (x) clinical evaluation of interventions within occupational therapy and speech therapy. This may have been due to a scarcity of research groups dedicated to these fields in Brazil.

**Figure 1.**
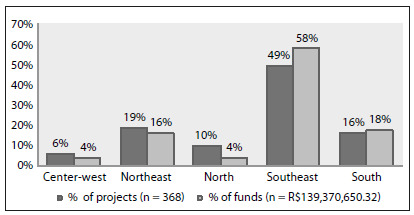
Investments by the Ministry of Health and its partners in clinical investigations and infrastructure for clinical research, according to geographical region, Brazil, 2002-2009.

**Figure 2.**
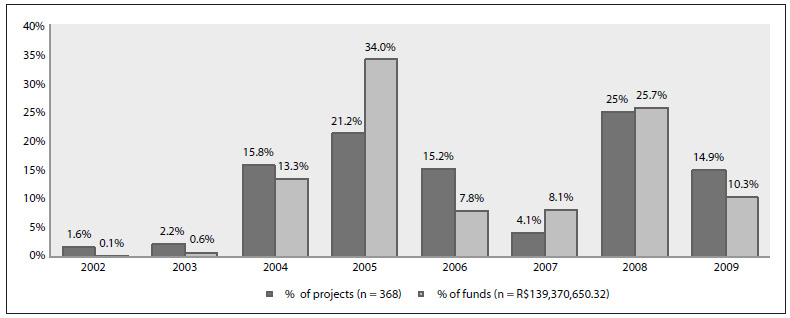
Annual investments made by the Ministry of Health and its partners in clinical investigations and infrastructure for clinical research, Brazil, 2002-2009.

Figure 1 shows the proportions of projects distributed according to geographical region; the southeastern region had the greatest proportion of the projects (49%) and financial resources (58%). This can be explained by the greater research tradition and larger number of qualified investigators in this region. Figure 2 shows the trend over the years. The higher proportion of projects in 2005 correlated with the creation of the RNPC, which had the mission of decentralizing clinical research in Brazil, among other objectives.

Another point worth noting is that the RNPC provides the opportunity to train new investigators. During the period analyzed, the 368 projects financed by the Ministry of Health and its partners contributed 575 high-level professionals trained in clinical investigation at postgraduate level. These funds made it possible to produce 373 master dissertations and 203 doctoral theses, concluded between 2002 and 2009.

## DISCUSSION

According to Zago, clinical research is an important tool for decision-making and aids in formulating health policies. However, Brazil needed to acquire its own data in order to formulate national public policies and solve the country’s specific problems.^15^ Not coincidentally, the expansion in clinical research matches the actions taken by the Ministry of Health regarding financing for infrastructure and organizing the RNPC. The creation of the RNPC demonstrates the government’s commitment to developing health science and technology activities in this country. Development of a network followed the constitutional guidance based on the three principles of universality, comprehensiveness and equity. It required effective involvement of clinical research teams with priorities as established in the National Health Plan.^2^

Neglected tropical diseases are primarily infectious diseases that thrive in impoverished settings, especially in the heat and humidity of tropical climates. More than one billion people, representing one-sixth of the world population, are infected with one or more of these diseases, which usually have very low priority in national healthcare programs.^16^ In Brazil, however, they deserve special attention from researchers and healthcare policymakers.^17^ The RNPC supports the clinical trials necessary for producing new national pharmaceuticals, principally those relating to neglected diseases. It is very important for the RNPC to promote scientific knowledge, thus meeting the demands of public concerns.^17^

In 2007 and 2008, the Ministry of Health, together with the MCT, financed clinical research projects to be carried out in institutions belonging to the RNPC, through public bidding, emphasizing those relating to the following topics: diabetes, cardiovascular disease, obesity, leishmaniasis, sleep apnea, osteoporosis and leprosy.

Many projects have already achieved interesting results. Recently, a clinical trial supported by the Ministry of Health showed promising results relating to the treatment of dermatological manifestations of Leishmania braziliensis infection. This randomized clinical trial indicated that after six months of treatment, the oral drug was more effective than the traditional antimony injections, with regard to clinical remission and treatment compliance, although having a similar incidence of side effects.^18^

Another study, conducted in Minas Gerais, focused on finding genetic polymorphisms associated with Chagas myocardiopathy. It found an association between interleukin-10 (IL-10) 1082G/A polymorphism and susceptibility to Chagas cardiomyopathy. This polymorphism was correlated with lower expression of IL-10, which was associated with worse cardiac function, as determined by left ventricular ejection fraction values in the study.^19^

A preclinical trial supported by the Ministry of Health showed that a protein derived from Carica candamarcensis in experimental rodent models presented mitogenic and healing properties.^20^ These proteins were previously patented by the research group as mitogenic proteases. Currently, the Ministry of Health is continuing to support the clinical stages of this study.

During the 2009 influenza A pandemic, the Ministry of Health decided to invest in strategic research in order to face up to the sanitary emergency better. Seven projects were financed, including investments in essential innovations for surveillance of influenza A. Most of the projects have already produced results. Initially, Brazil, as well as most countries, was totally dependent on inputs and reagents obtained from the United States Centers for Disease Control for the diagnostic kits. However, the “Nationalization of biotechnological products for molecular diagnosis of influenza A (H1N1)” project was financed in 2009 and, six months later, the investigators launched the National Diagnostic Kit, thereby promoting access to inputs from molecular biology to achieve diagnostic tests for the influenza A pandemic virus. Moreover, biotechnological platforms have been deployed in three key national reference laboratories for influenza and three public health laboratories; equipment maintenance and human resources technical training has also been ensured.^21^

The results described here demonstrate the Ministry of Health’s progressive advances as a sponsor and key stakeholder in implementation and promotion of clinical research directed towards Brazil’s national requirements, largely due to creation of the RNPC. Future healthcare policies should promote democratization of the clinical research funding processes in order to decentralize research investments throughout the country. Currently, many clinical studies are taking place through the RNPC and their conclusions will help to improve the Brazilian public health system.

Recently, a study showed how different research fields are prioritized for NIH (National Institutes of Health) support.^22^ The authors concluded that, in 2006, the levels of disease-specific funding correlated only modestly with the burden of disease in the United States, and the correlation had not improved in relation to 1996 levels. The conditions receiving the most funding were AIDS and diabetes while on the other hand, the least amount of funding, relative to the burden of disease, was destined for depression and injuries.^22^

Funding for practice-oriented clinical research in the United Kingdom was considered insufficient in 2006.^23^ Biomedical and etiological research, which was mostly laboratory-based, spent from 60% to 90% of the budget allocations from four major funding institutions in the United Kingdom. Important topics such as prevention, detection and diagnosis, treatment development and evaluation, disease management and health service research received only a small fraction of the funds.^23^ More recently, another author expressed the opinion that overregulation of clinical research in Britain had led to decreased numbers of ongoing clinical trials.^24^ The British National Institute for Health Research had built a sustainable infrastructure to support clinical research for patients’ benefit. However, while the safety and interests of patients were protected, clinical research in the United Kingdom had become unnecessarily stifled by incremental bureaucratic arrangements. The British Academy of Medical Sciences was consulted to make recommendations with the aim of optimizing clinical research. They suggested that a National Research Governance Service, responsible for centralizing ethical reviews of protocols and project monitoring, should be created.^24^

In some respects, clinical research in Brazil compares favorably with the situation in other BRICS countries. Clinical research in Russia was on the rise over the last decade, thus leading to optimism about attainment of Russia’s potential to undertake clinical trials.^25^ However, in 2007, the Federal Customs Service completely blocked the exportation of all human biological materials from Russia, from hair to tissue and blood samples, thereby threatening dozens of clinical trials, under the suspicion of “involvement of Western institutions in the development of genetic weapons against Russia”.^26^

Serious concerns have been raised about ethical issues relating to clinical trials conducted in India and the local ethics committees’ lack of independence.^27^^,^^28^ A recent investigation revealed at least four trials, run by large pharmaceutical companies, which violated the Indian Medical Research Council’s ethical guidelines and the World Medical Association’s Declaration of Helsinki.^28^ With a huge population, a plethora of diseases and rampant poverty, India is considered to be an “irresistible” location for clinical trials.^29^ Pharmaceutical industry giants are attracted to India because of its 700,000 specialty hospital beds, 221 medical colleges and skilled English-speaking medical personnel. However, the biggest advantage is the low cost: trials for a standard drug in the United States can cost about $150 million and a similar drug could be tested in India at a 60% lower cost.^29^

The status of clinical research in China was recently reviewed.^30^ The authors showed that the numbers of clinical trials and published papers in global first-class journals had increased over the period under review (2000-2009). The number of RCTs conducted in China increased from 85 in 2000 to 743 in 2009, and constituted nearly a third of the total number of published clinical research papers in China. Out of the 2,500 Chinese physicians interviewed, only a minority had reasonable knowledge about clinical research. However, most of them mentioned that there was a need for research in this field and expressed willingness to participate in clinical trials. The authors concluded that China still needed to step up its efforts in many respects, in order to promote the development of clinical research.^30^

From South Africa, there has been a report on how the lack of investment in the field reduced the number of projects running and articles published between 1980 and 2009.^31^ The South African Medical Research Council, which falls under the country’s Department of Health, has failed so far to link itself to these important and highly effective investments.^31^ A survey on how to improve clinical research was conducted, based on interviews with senior decision-makers at institutions with stakes in the South African public sector clinical trial research environment.^32^ There was a consensus regarding the need to establish a sustainable national clinical trial support initiative. An agency with this mission should be responsible for funding, staffing, making quality controls, monitoring and overseeing approved clinical trials.^32^

One successful experience in this direction was the establishment of the National Clinical Research Network in Brazil. A leading Brazilian scientist in this field stated that: “The leadership exerted by the Ministry of Health with regard to equipping teaching hospitals to develop quality clinical research is clear. The creation of the RNPC, with funding for infrastructure and development of appropriate multicenter projects of interest to SUS, has been contributing decisively towards recognition and institutionalization of clinical research in this country’s teaching hospitals”.^2^

## CONCLUSION

The Ministry of Health’s strategy of creating the RNPC brought together the partners needed to build a new model for clinical research, dedicated to the real needs of SUS. Through seeking to consolidate this network, priority has been given to standardization and implementation of all phases of clinical trials on drugs, procedures, equipment, medical devices and new diagnostic methods. The “PesquisaSaúde” research management database is an important tool for registering the government’s actions, thereby ensuring that the research is completely accessible to all those involved in healthcare decision-making, with improved research transparency and strengthening of the value of evidence-based decisions. The investments have ensured that clinical research in Brazil has been strengthened, through adequate infrastructure, researchers with highly professional qualifications and an increased link between teaching and research. Ministry of Health actions have ensured cohesion between health policies, knowledge production and the healthcare industrial sector.
